# Therapeutic Treatment of Arthritic Mice with 15-Deoxy Δ^12,14^-Prostaglandin J_2_ (15d-PGJ_2_) Ameliorates Disease through the Suppression of Th17 Cells and the Induction of CD4^+^CD25^−^FOXP3^+^ Cells

**DOI:** 10.1155/2016/9626427

**Published:** 2016-10-31

**Authors:** Vanessa Carregaro, Marcelo H. Napimoga, Raphael S. Peres, Luciana Benevides, Laís Amorim Sacramento, Larissa G. Pinto, Renata Grespan, Thiago M. Cunha, João Santana da Silva, Fernando Q. Cunha

**Affiliations:** ^1^Department of Biochemistry and Immunology, School of Medicine of Ribeirão Preto, 14049-900 Ribeirão Preto, SP, Brazil; ^2^Laboratory of Immunology and Molecular Biology, São Leopoldo Mandic Institute and Research Center, 13045755 Campinas, SP, Brazil; ^3^Department of Pharmacology, School of Medicine of Ribeirão Preto, 14049-900 Ribeirão Preto, SP, Brazil

## Abstract

The prostaglandin, 15-deoxy Δ^12,14^-prostaglandin J_2_ (15d-PGJ_2_), is a lipid mediator that plays an important role in the control of chronic inflammatory disease. However, the role of prostanoid in rheumatoid arthritis (RA) is not well determined. We demonstrated the therapeutic effect of 15d-PGJ_2_ in an experimental model of arthritis. Daily administration of 15d-PGJ_2_ attenuated the severity of CIA, reducing the clinical score, pain, and edema. 15d-PGJ_2_ treatment was associated with a marked reduction in joint levels of proinflammatory cytokines. Although the mRNA expression of ROR-*γ*t was profoundly reduced, FOXP3 was enhanced in draining lymph node cells from 15d-PGJ_2_-treated arthritic mice. The specific and polyclonal CD4^+^ Th17 cell responses were limited during the addition of prostaglandin to cell culture. Moreover,* in vitro* 15d-PGJ_2_ increased the expression of FOXP3, GITR, and CTLA-4 in the CD4^+^CD25^−^ population, suggesting the induction of Tregs on conventional T cells. Prostanoid addition to CD4^+^CD25^−^ cells selectively suppressed Th17 differentiation and promoted the enhancement of FOXP3 under polarization conditions. Thus, 15d-PGJ_2_ ameliorated symptoms of collagen-induced arthritis by regulating Th17 differentiation, concomitant with the induction of Tregs, and, consequently, protected mice from diseases aggravation. Altogether, these results indicate that 15d-PGJ_2_ may represent a potential therapeutic strategy in RA.

## 1. Introduction

Rheumatoid arthritis (RA) is a chronic disorder characterized by chronic systemic inflammation and progressive destruction of cartilage and bone. The etiology of RA is unknown, but proinflammatory cytokines play a central role in the disease development and perpetuation [1]. Among several cytokines, IL-17 is expressed in the synovial tissue of RA patients and animals models and had been implicated in the initiation and progression of arthritis [2]. In murine arthritis models, IL-17 promotes the activation of synovial fibroblasts and both leukocyte emigration and activation, resulting in the production of several inflammatory mediators and tissue lesions. For example, IL-17 has been shown to enhance joint inflammation and the tissue production of cytokines (TNF-*α*, IL-1*β*) [3], chemokines (MIP-2/CXCL2, KC/CXCL1, and IL-8/CXCL8), and matrix metalloproteinases [4]. Given the ability of IL-17 to promote RA pathology, it is plausible to suggest that pharmacologic strategies aimed at blocking or suppressing IL-17, particularly cellular Th17 function, may deserve attention as a potential therapeutic strategy for autoimmune diseases.

The current treatments for RA are scarce and only provide symptomatic relief with limited effects on the progression of the disease. Thus, additional new therapies are needed [5]. Although Peroxisome Proliferator-Activated Receptor-*γ* (PPAR-*γ*) is a master transcriptional regulator of adipocyte differentiation, the anti-inflammatory activity of this receptor is also well described [6]. PPAR-*γ* modulates T cell activity by inhibiting IL-2 production in T cell receptor-stimulated Th cells [7] and by suppressing Th2 cell activity [8]. Moreover, previous studies demonstrated that PPAR-*γ* is an intrinsic suppressor for Th17 cell generation [9, 10]. PPAR-*γ* activation is thought to prevent the removal of repressor complexes from the ROR-*γ*t gene promoter, thus suppressing ROR-*γ*t expression and Th17 cell differentiation in an intrinsic manner. Moreover, multiple sclerosis patients are highly susceptible to PPAR*γ*-mediated suppression of Th17 cell development, strongly asserting PPAR-*γ* as a promising target for specific immunointervention in autoimmune disorders [9]. Therefore, PPAR-*γ* ligands, including endogenous and synthetic agonists such as linoleic acid, 15-deoxy-Δ^12,14^-prostaglandin J_2_ (15d-PGJ_2_), and thiazolidinediones, have extensive potential in the treatment of chronic inflammatory diseases [11–13]. Therefore, we examined the potential therapeutic effect of the natural PPAR-*γ* agonist, 15d-PGJ_2_, on collagen-induced arthritis (CIA).

## 2. Methods

### 2.1. Mice

Male DBA/1J mice weighing 18–22 g were housed at the animal facility of the Department of Pharmacology or Immunology, School of Medicine of Ribeirão Preto, University of São Paulo (Brazil), in temperature-controlled rooms (22–25°C), and received water and food ad libitum. All experiments were conducted in accordance with the National Institutes of Health (NIH) guidelines on the welfare of experimental animals and with the approval of the Ethics Committee from the School of Medicine of Ribeirão Preto.

### 2.2. Induction of CIA and Assessment of Arthritis

CIA was elicited in mice as previously described [14, 15]. Briefly, male DBA/1J mice (10 wk) received 200 *μ*g bovine type II collagen (C-II) (Sigma) diluted in acetic acid and emulsified in Freund's complete adjuvant (Sigma) by intradermal (i.d.) injection at the base of the tail on day 0. Mice were boosted i.d. with collagen (200 *μ*g diluted in acetic acid) emulsified in Freund's incomplete adjuvant (Sigma) on day 21. Mice were monitored daily for signs of arthritis as described [14, 15]. Scores were assigned based on erythema, swelling, or loss of function present in each paw on a scale of 0–3, resulting in a maximum score of 12 per mouse. When mice reached a score of 1 for clinical arthritis, they were treated with 15d-PGJ_2_ (1 mg/kg) by the s.c. route daily for 7 days. Control mice received the same volume of PBS. Scoring was conducted in a blinded fashion. Mechanical hypernociception (pain) evaluation in the tibia-tarsal joint was performed with an electronic anesthesiometer (model 1601C, Life Science Instruments, California, USA) consisting of a pressure transducer connected to a digital counter force in grams (g). Capture is achieved by pressure contact of the paw pressure transducer, which is accomplished through a polypropylene tip area that is connected to the transducer. The assessment of nociception in the tibia-tarsal joint consists of the application of increasing pressure on the paw of the mouse through mesh until the animal flexes the femur and tibia, producing a paw withdrawal response. The intensity of mechanical hypernociception of the joint is measured by the absolute values of the mechanical threshold (in grams). For verification of edema, paw thickness was measured daily using a caliper, and the values are expressed in millimeters (mm). For histologic assessment, mice were euthanized 35 days after challenge, and the hind limbs were removed and demineralized thoroughly in 10% EDTA for 1-2 wk. The decalcified tissues were trimmed, dehydrated in graded ethanol, and embedded in paraffin. Serial sections (5 *μ*m) were cut and mounted on glass slides precoated with 0.1% poly-L-lysine (Sigma). Histologic assessment was performed following routine hematoxylin and eosin staining (H&E). Ankle and joint sections were prepared and stained with H&E to study the inflammatory cell influx or using safranin-O to determine proteoglycan depletion and cartilage destruction. To measure cytokine concentrations in the inflammatory site, articular tissues were harvested, weighed, and titered in 1 mL of PBS containing complete protease inhibitor cocktail (Roche) by a tissue trimmer. Articular homogenates were centrifuged, and the supernatants were collected and stored at −70°C for determination of IFN-*γ*, IL-12, IL-17, and TNF-*α* levels by ELISA (BD Biosciences), according to the manufacturer's instructions.

### 2.3. T Cell Proliferation

To assess the influence of 15d-PGJ_2_ treatment on T cell proliferation, popliteal and inguinal lymph nodes cells harvested from arthritic mice were removed and washed twice with PBS. Tissues were minced, and the cells were filtered through a cell strainer, centrifuged at 500 ×g at 4°C for 10 min, and resuspended in RPMI-1640 medium at 2.5 × 10^6^ cells/mL. In some wells, cells were incubated with 15d-PGJ_2_ (5 *μ*M) or vehicle (DMSO 0.5%) 1 hour before stimulation. In all of the experiments, C-II (5 *μ*g/mL), plate-bound anti-CD3 mAb (5 *μ*g/mL), or medium was added to the culture and incubated for 96 h in a total volume of 200 *μ*L per condition. Supernatants were harvested for determination of IL-17 production using ELISA, and cell proliferation was measured by overnight [^3^H]thymidine incorporation.

### 2.4. Flow Cytometry

Popliteal and inguinal lymph nodes from arthritic mice were harvested 7 days after arthritis symptoms and processed, and cells were cultured with 15d-PGJ_2_ (5 *μ*M) or vehicle (PBS DMSO 0.05%) 1 hour before anti-CD3 mAb stimulation. Cells were incubated with fluorochrome-conjugated mAb anti-CD4, anti-CD25, CTLA-4, and GITR for 30 min at 4°C, washed, and fixed with BD Cytofix (BD Biosciences). Cells were permeabilized using PBS containing 1% FCS, 0.01% sodium azide, and 0.05% saponin and stained with anti-mouse FOXP3 (all antibodies from BD Biosciences), acquired on FACS Canto II (BD Biosciences), and analyzed using FlowJo software (Tree Star).

### 2.5. Generation of Th17 Cells and Regulatory T Cells

CD4^+^CD25^−^ or CD4^+^CD25^+^ cells from the spleen were isolated using a CD4^+^CD25^+^ regulatory T cell kit (Miltenyi Biotec, Auburn, CA) in accordance with the manufacturer's instructions, and a purity of ~95% was obtained for each T cell subset. For Th17 differentiation, CD4^+^CD25^−^ cells (5.0 × 10^5^ cells/well) were stimulated with plate-bound anti-CD3 mAb (5 *μ*g/mL), anti-CD28 mAb (1.0 *μ*g/mL), rmTGF-*β* (2.5 *η*g/mL), IL-6 (10 *η*g/mL), and anti-IFN-*γ* mAb (10 *μ*g/mL). For Treg differentiation, CD4^+^CD25^−^ cells (5.0 × 10^5^ cells/well) were stimulated with rmTGF-*β* (5 ng/mL), rmIL-2 (100 U/mL), anti-IFN-*γ* (10 *μ*g/mL), and anti-IL-4 (10 *μ*g/mL). In all experiments, 15d-PGJ_2_ (5 *μ*M) or medium was added to the culture on days 0, 3, and 5, and the cells were incubated at 37°C in 5% CO_2_ for 7 days in a total volume of 200 *μ*L per condition. As a differentiation control, nTreg (CD4^+^CD25^+^) or Th0 (CD4^+^CD25^−^) cells were cultured in the presence of IL-2 (100 U/mL) for T cell maintenance. The lymphocytes were then washed and phenotyped for the expression of surface markers using monoclonal antibodies specific for CD4 or CD3 conjugated to FITC or PerCP (BD & Biosciences eBioscience, San Diego, CA, USA). For intracellular IL-17 or FOXP3, stained cells were washed twice with PBS and centrifuged at 400 ×g for 10 minutes, followed by incubation with Cytofix/Cytoperm (BD Biosciences) for 15 minutes. Samples were again washed and incubated with a specific antibody for IL-17 conjugated to PE diluted in 1x Permwash for 10 min and after further washing in PBS were acquired on a FACSCanto II unit (BD Biosciences). Analyses were performed using FlowJo software (TreeStar, Ashland, OR, USA).

### 2.6. Quantitative RT-PCR

Total RNA was extracted from draining lymph nodes (inguinal and popliteal) of naïve or arthritic animals treated with 15d-PGJ_2_ using RNAspin Mini Isolation Kit (GE Healthcare, Buckinghamshire, Germany) following the manufacturer's recommendations. Gene expression was normalized to the expression of the GAPDH gene: GAPDH forward: 5′-TGCAGTGGCAAAGTGGAGAT-3′; reverse: 5′-CGTGAGTGGAGTCATACTGGAA-3′; PPAR-*γ* forward 5′-TGAGATCATCTACACGATGCT-3′; reverse: 5′-GGAACTCCCTGGTCATGAA-3′; ROR-*γ*t forward 5′-GCTTCCCAATGGACACTTGCAAG-3′; and reverse: 5′-AGGACAGCACACAGCTGGCAGTGG-3′; FOXP3 forward: 5′-ACAACCTGAGCCTGCACAAGT-3′; reverse: 5′-GCCCACCTTTTCTTGGTTTTG-3′.

### 2.7. Statistical Analysis

Data are expressed as the mean ± SEM and are representative of 2–4 independent experiments. The results of individual experiments were not combined, as they were analyzed individually. The means from different groups were compared by analysis of variance (ANOVA) followed by Tukey's test. Statistical significance was set at *P* < 0.05.

## 3. Results

### 3.1. Therapeutic Effect of 15d-PGJ_2_ on the Development of Experimental Rheumatoid Arthritis

**Figure 1:**
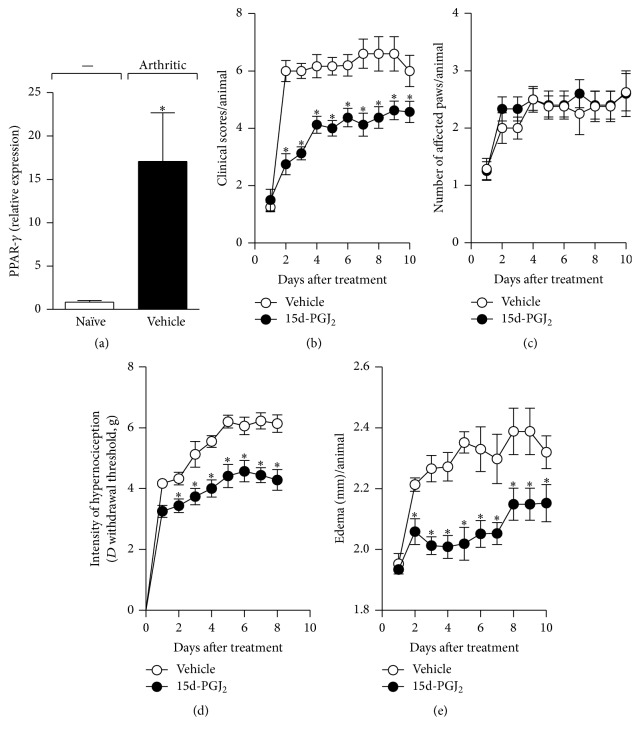
15d-PGJ_2_ attenuated collagen-induced arthritis. PPAR-*γ* mRNA expression was quantified by real-time PCR in draining lymph nodes from naïve (white bar) or collagen-immunized and challenged DBA/1 (black bar) mice on the seventh day of disease (a). Arthritic mice were treated by the subcutaneous route with vehicle (○) or 15d-PGJ_2_ (1 mg/Kg) (●) for 7 d. Mice were monitored for disease progression as indicated by clinical scores (b), number of affected paws (c), hypernociception (d), and edema (e). Results are presented as the mean ± SEM,* N* = 8–10; ^*∗*^ *P* < 0.05 compared with the PBS-treated group.

PPAR-*γ* is a potent modulator of inflammatory responses [16, 17]. We investigated whether PPAR-*γ* is expressed during collagen-induced arthritis (CIA), a murine model that shares similarities with rheumatoid arthritis (RA). CIA was elicited in DBA/1J mice, as described in Section 2, and draining lymph nodes (inguinal and popliteal) from naïve or arthritic animals were harvested 7 days after disease manifestation. As shown in Figure 1(a), the PPAR-*γ* mRNA transcript was highly expressed in the lymph nodes of arthritic animals when compared with the control group (naive animals). Next, the potential therapeutic effect of the PPAR-*γ* agonist, 15d-PGJ_2_, on CIA was evaluated. Mice were treated daily with 15d-PGJ_2_ (1 mg/kg) by the subcutaneous route for 7 days from the first day of clinical manifestation of disease. Controls received vehicle (PBS). As expected, control mice (vehicle-treated) developed a severe disease from day 22 until day 30 after CIA induction, exhibiting high clinical scores (Figure 1(b)), mechanical hypernociception (Figure 1(d)), and edema (Figure 1(e)) (2.43 ± 0.12). However, the treatment of arthritic mice with 15d-PGJ_2_ attenuated the severity of the disease, with a reduction in the clinical scores (Figure 1(b)), mechanical hypernociception (Figure 1(d)), and swelling (Figure 1(e)). With respect to the numbers of affected paws, no significant difference was observed between the groups (15d-PGJ_2_ and vehicle) (Figure 1(c)), suggesting that prostanoid treatment interfered with progression but did not prevent disease onset (see Table 1 in Supplementary Material available online at http://dx.doi.org/10.1155/2016/9626427).

**Figure 2:**
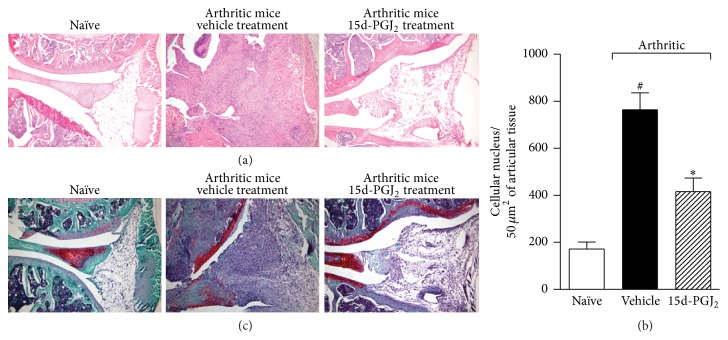
15d-PGJ_2_ treatment ameliorates articular inflammation. Naïve or collagen-immunized and challenged DBA/1 mice were injected s.c. daily with vehicle or 15d-PGJ_2_ (1 mg/Kg) for 7 days. At the end of treatment, mice were euthanized, the articular joints were harvested, and histopathologic analysis was performed. Knee joint sections were stained with H&E (a) or with safranin-O (c), a proteoglycan red marker, to reveal profound cartilage damage in the vehicle-treated mice (less proteoglycan staining) and the preservation of cartilage in 15d-PGJ_2_-treated mice. Quantification of cellular infiltrate was performed by ImageJ software (NIH, USA) in 40 fields with 400x magnification for each animal/group (b). Morphometric histologic examination revealed markedly less cellular infiltration in the 15d-PGJ_2_ mice than in the PBS-treated group. ^#^ *P* < 0.05 compared with naïve group. ^*∗*^ *P* < 0.05 compared with arthritic mice treated with PBS (vehicle).

Histologic analyses of the knees at the end of the monitoring period revealed that untreated arthritic mice exhibited severe cellular infiltration (Figures 2(a) and 2(b)) and marked reductions in matrix proteoglycan (Figure 2(c)), suggesting joint cartilage damage. In contrast, these pathologic events were reduced in 15d-PGJ_2_-treated animals (Figure 2(b)). Altogether, these data suggest that 15d-PGJ_2_ attenuated the severity of CIA and prevented the progression of articular damage.

### 3.2. 15d-PGJ_2_ Treatment Reduces Proinflammatory Cytokine Production

**Figure 3:**
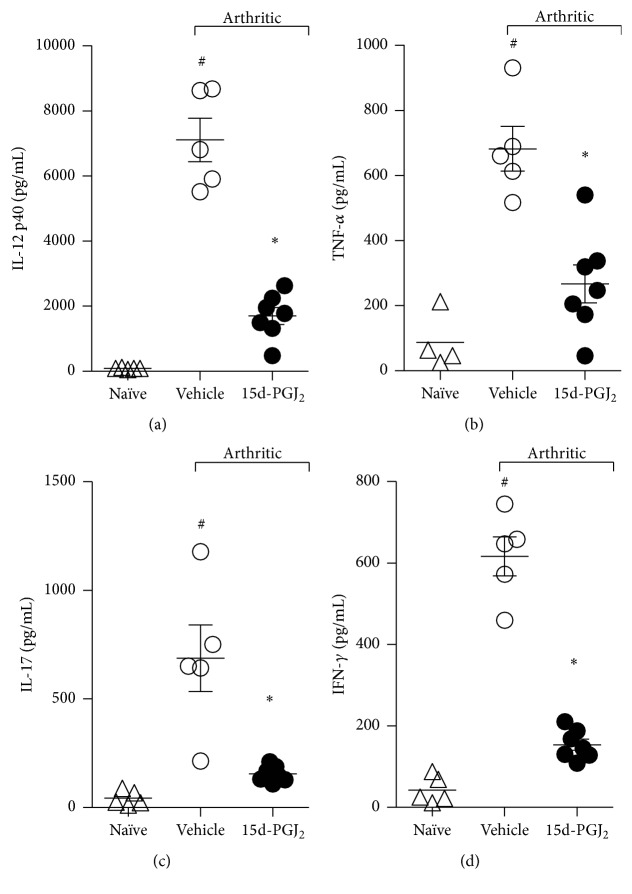
Decreased inflammatory cytokines in articular joints from 15d-PGJ_2_-treated arthritic mice. Ankle joints from naïve (Δ) or PBS- (○) or 15d-PGJ_2_-treated (●) arthritic mice were collected after 7 days of treatment for the determination of TNF-*α* (b), IFN-*γ* (d), IL-17 (c), and IL-12 (a) levels by ELISA in the homogenate supernatants. Results are expressed as the mean ± SEM,* N* = 4 (naïve) and 9-10 (arthritic groups). ^#^ *P* < 0.05 compared with naïve group. ^*∗*^ *P* < 0.05 compared with arthritic mice treated with PBS (vehicle).

Given that the onset and progression of autoimmune diseases (including rheumatoid arthritis (RA)) are mediated by proinflammatory cytokines released into the inflammatory site, we investigated the effect of 15d-PGJ_2_ treatment upon the production of TNF-*α*, IFN-*γ*, IL-17, and IL-12 in affected ankle joints. Paw samples from arthritic mice treated with vehicle (PBS) contained significantly higher concentrations of all abovementioned inflammatory cytokines compared with those of naïve mice (Figure 3). However, mice treated with 15d-PGJ_2_ exhibited a significant reduction in the levels of IL-12 (Figure 3(a)), TNF-*α* (Figure 3(b)), IL-17 (Figure 3(c)), and IFN-*γ* (Figure 3(d)) compared with vehicle-treated arthritic mice.

### 3.3. 15d-PGJ_2_ Suppresses the Inflammatory Response

**Figure 4:**
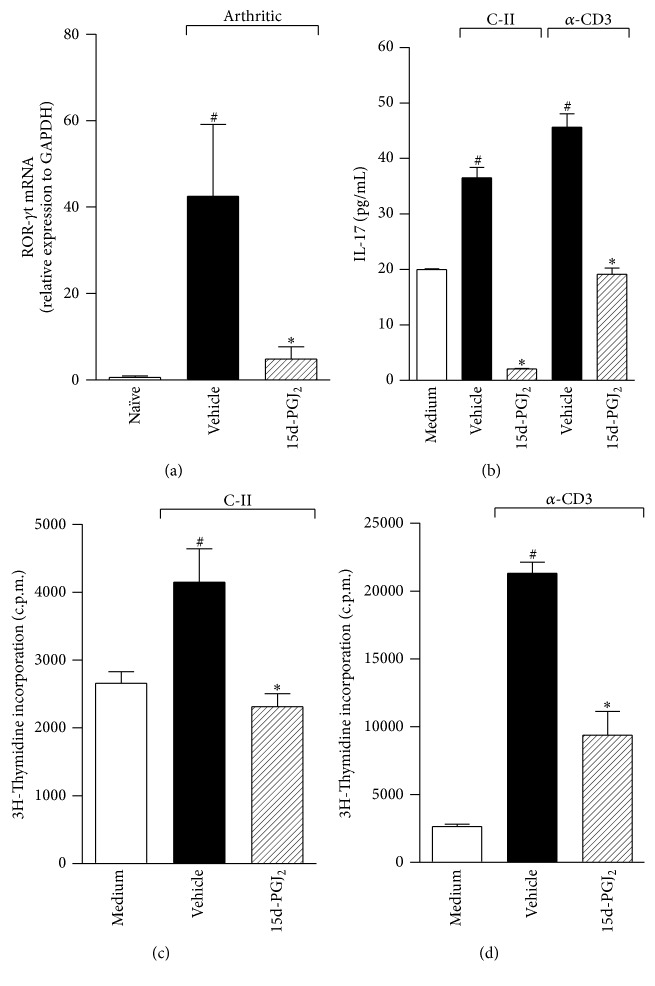
15d-PGJ_2_ suppresses the collagen-induced Th17 immune response. ROR-*γ*t mRNA expression was quantified by real-time PCR in draining lymph nodes from naïve mice (white bar) or collagen-immunized and challenged DBA/1 mice treated with vehicle (PBS) (black bar) or – 15d-PGJ_2_ (1 mg/Kg) (hatched bar) for 7 days (a). ^#^ *P* < 0.05 when compared with naïve mice. ^*∗*^ *P* < 0.05 when compared with vehicle (PBS). 15d-PGJ_2_-pretreated nonadherent cells (2 × 10^6^ cells/mL) (1 hour before) from draining LNs from the mice above were stimulated* in vitro* with C-II (5 *μ*g/mL) or plate-bound *α*-CD3 (5 *μ*g/mL) for 96 h. Culture supernatants were harvested to measure IL-17 (b) levels from C-II- or *α*-CD3-stimulated cultures. The specific C-II (c) or *α*CD3 polyclonal (d) stimuli proliferation assay was assessed by overnight [^3^H]thymidine incorporation. The results are expressed as the mean ± SEM obtained from triplicate samples from two or three independent experiments (*N* = 3 per group). ^#^ *P* < 0.05 when compared with medium. ^*∗*^ *P* < 0.05 compared with vehicle (PBS).

Given that 15d-PGJ_2_ attenuated the severity of arthritis (Figure 1), we investigated whether treatment with this prostanoid interferes with the pattern of the Th17 response. First, we analyzed the mRNA levels of the common Th17 transcription factor, ROR-*γ*t. RT-PCR analyses revealed that the expression of ROR-*γ*t was increased in arthritic animals. Interestingly, the transcript for ROR-*γ*t was decreased when arthritic animals were treated with 15d-PGJ_2_ (Figure 4(a)). To investigate the impact of 15d-PGJ_2_ treatment upon the collagen-specific response, draining lymph node CD4^+^ T cells from arthritic mice were sorted and treated for 1 hour with prostanoid (5 *μ*M). The proliferative response and IL-17 levels were measured in the culture. Using different culture conditions with specific antigen (collagen) or soluble anti-CD3 antibody for 96 hours, higher levels of IL-17 were detected relative to the medium alone. However, the addition of 15d-PGJ_2_ ablated IL-17 production even under polyclonal stimulation (Figure 4(b)). Prostanoid treatment also suppressed the proliferative immune response induced by the specific (Figure 4(c)) or polyclonal (Figure 4(d)) stimuli relative to vehicle. These data demonstrate the immunosuppressive effect of 15d-PGJ_2_ in the inflammatory immune response during CIA.

### 3.4. 15d-PGJ_2_ Promotes the Treg Profile among Effector T Cells

**Figure 5:**
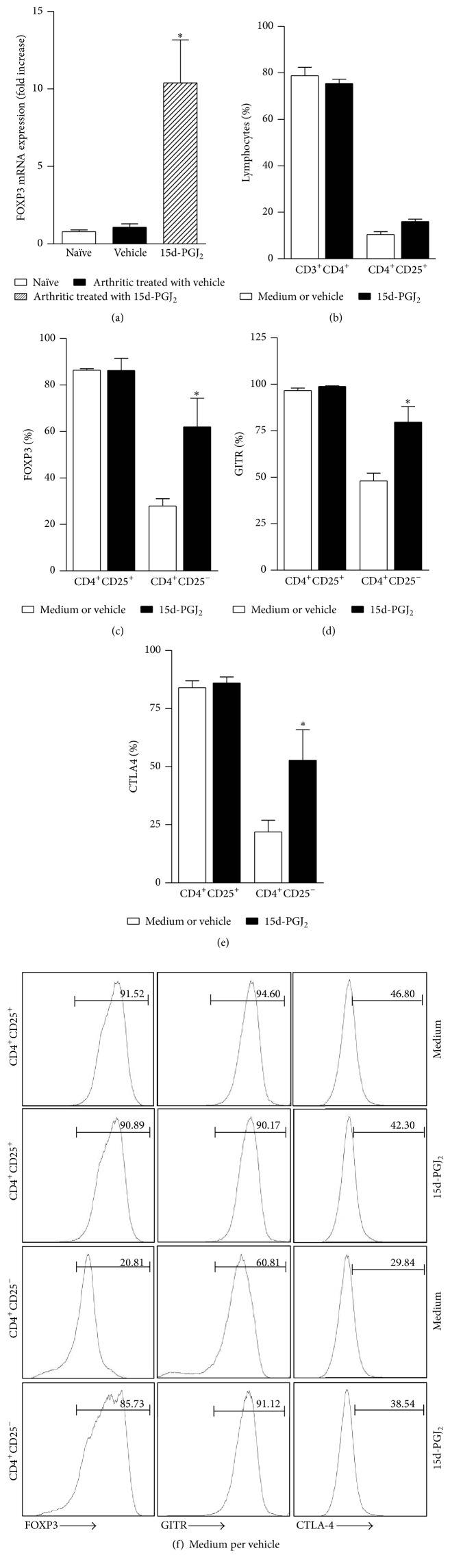
15d-PGJ_2_ induces regulatory T cell markers in conventional T cells. (a) FOXP3 mRNA expression was quantified by real-time PCR in draining lymph nodes from naïve mice (white bar) or collagen-immunized and challenged DBA/1 mice treated with vehicle (black bar) or 15d-PGJ_2_ (hatched bar) after 7 days of treatment. Results are presented as the mean ± SEM, *N* = 6; ^*∗*^ *P* < 0.05 compared with PBS-treated group. Total cells (2 × 10^6^ cells/mL) from the draining lymph nodes from naïve or arthritic animals were* in vitro* incubated with 15d-PGJ_2_ (5 *μ*M) (black bars) or vehicle (DMSO) (white bars) for 96 hours on plates coated with *α*-CD3. The nonadherent cells were phenotyped by flow cytometry using specific antibodies: anti-CD3 conjugated with FITC, anti-CD4 conjugated with PerCP, and anti-CD25 conjugated with APC-Cy7 (b) and anti-FOXP3 (c), anti-GITR (d), and anti-CTLA-4 (e) conjugated with PE. Lymphocytes were gated on CD4^+^CD25^+^ or CD4^+^CD25^−^, and the population expressing the markers described above was subsequently analyzed. In (f), representative histograms of FOXP3, CTLA-4, and GITR are shown in each box. The values above are expressed as the mean ± SEM, which are representative of quadruplicate samples from two independent experiments (*N* = 4). ^#^ *P* < 0.05 compared with CD4^+^CD25^−^ group control (vehicle).

To further explore the immunomodulatory effect of 15d-PGJ_2_ on CIA, we evaluated the expression of FOXP3, a transcription factor highly expressed in regulatory T cells that is related to the control of the immune response both* in vitro* and* in vivo* [18]. Interestingly, the induction of arthritis in mice did not increase FOXP3 mRNA expression in the draining lymph nodes of animals that received vehicle as treatment (Figure 5(a)). However, FOXP3 was highly expressed in the lymph nodes of arthritic animals treated with 15d-PGJ_2_, and this increase was sixfold higher than the group of arthritic animals treated with vehicle.

To examine whether 15d-PGJ_2_ affects FOXP3 expression in nTreg cells, we examined CD4^+^ T cells after* in vitro* incubation with 15d-PGJ_2_ (5 *μ*M) and restimulation with collagen or plate-bound *α*-CD3. As shown in Figure 5(b), treatment with 15d-PGJ_2_ did not interfere with CD3^+^CD4^+^ positivity compared to the vehicle. Similar effects were observed concerning the CD4^+^CD25^+^ population (Figure 5(b)). The expression of Treg markers like FOXP3 (Figure 5(c)), GITR (Figure 5(d)), and CTLA-4 (Figure 5(e)) in the CD4^+^CD25^+^ cells was also similar to those recovered from both the culture with the prostanoid and the culture with the vehicle. Unexpectedly, we detected the expression of markers characteristic of Tregs on the gate of CD4^+^CD25^−^, described as conventional T cells, and we observed a significant increase in the expression of FOXP3 (Figure 5(c)), GITR (Figure 5(d)), and CTLA-4 (Figure 5(e)), suggesting that 15d-PGJ_2_ induces a regulatory T cell phenotype in conventional T lymphocytes.

### 3.5. Effect of 15d-PGJ_2_ during T Cells Differentiation

**Figure 6:**
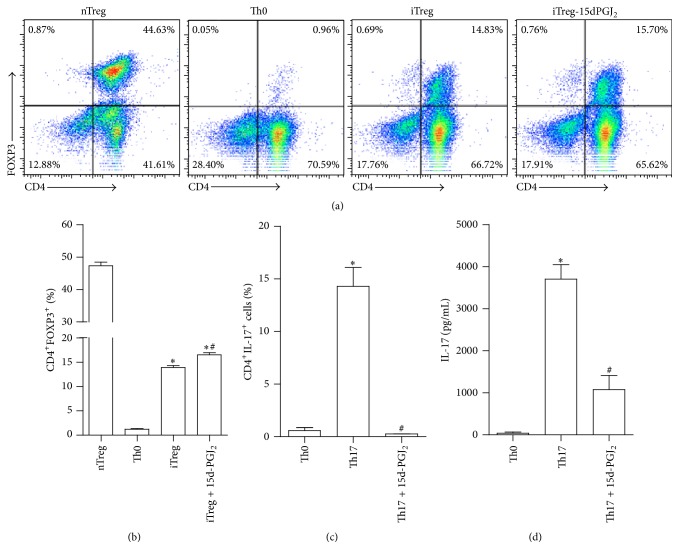
15d-PGJ_2_ altered the profile of CD4^+^CD25^−^ cells under polarizing conditions. Isolated CD4^+^CD25^−^ cells from naïve mice were cultured under Treg ((a)/(b)) or Th17 ((c)/(d)) polarizing conditions with or without 15d-PGJ_2_ (5 *μ*M). Natural Treg (nTreg, CD4^+^CD25^+^) or Th0 (CD4^+^CD25^−^) cells were used as positive (for Treg) and negative differentiation controls. The bars represent the percentage of TCD4^+^ cells expressing FOXP3 or IL-17. In (d), IL-17 levels were measured into supernatant culture from Th17 polarizing condition by ELISA assay. The results are expressed as the mean ± SEM obtained from triplicate samples from one of three independent experiments (*N* = 3 per group). ^*∗*^ *P* < 0.05 relative to the vehicle group. ^*∗*^ *P* < 0.05 compared with Th0; ^#^ *P* < 0.05 compared with iTreg (b). ^*∗*^ *P* < 0.05 compared with Th0; ^#^ *P* < 0.05 compared with Th17 (c-d).

Based on our finding that prostanoid treatment altered the phenotype of conventional T cells to a regulatory profile, additional experiments were conducted in purified CD4^+^CD25^−^ populations to obtain unequivocal evidence for the role of 15d-PGJ_2_ in induced Treg (iTreg) generation. To generate iTregs, sorted CD4^+^CD25^−^ T cells from DBA/J naïve mice were cultured on plate-bound anti-CD3 mAb with anti-CD28 mAb, rmTGF-*β*, rmIL-2, anti-IFN-*γ* mAb, and anti-IL-4 mAb in the presence or absence of 15d-PGJ_2_ (5 *μ*M) or vehicle for 7 d. At the end of culture period, the cells were harvested and analyzed for FOXP3 expression by flow cytometry. As control groups, natural regulatory T cells (CD4^+^CD25^+^) or Th0 (CD4^+^CD25^−^) were cultured only in the presence of rmIL-2 for cell maintenance. As expected, FOXP3 was highly expressed in nTregs (CD4^+^CD25^+^). Under Treg-polarizing conditions, FOXP3 was also expressed in CD4^+^CD25^−^ cells cultured with vehicle but was enhanced when 15d-PGJ_2_ was added to the culture (Figure 6(a)).

To further characterize the effect of prostanoid on Th17 differentiation, sorted naïve CD4^+^CD25^−^ T cells from DBA mice were cultured on plate-bound anti-CD3 mAb with anti-CD28 mAb, rmTGF-*β*, rmIL-1*β*, anti-IFN-*γ* mAb, anti-IL-4 mAb, and 15d-PGJ_2_ (5 *μ*M) or vehicle for 7 d. In the presence of 15d-PGJ_2_, CD4^+^CD25^−^ Th17 differentiation was strongly reduced (Figure 6(b)). It is important to note that this effect on Th17 culture was not due to a cytotoxicity effect, as propidium iodide (PI+) positivity was not observed at any concentrations of 15d-PGJ_2_ tested (Supplementary Figure 1). Altogether, the data suggest that 15d-PGJ_2_ may modulate iTreg generation and inhibit the Th17 subset differentiation.

## 4. Discussion

In the present study, we demonstrated that the expression of the PPAR-*γ* receptor is enhanced during experimental collagen-induced rheumatoid arthritis (CIA) and that its natural ligand, 15d-PGJ_2_, reduces the severity of RA, characterized by a decrease in clinical scores, joint hyperalgesia, and edema as well as leukocyte migration to the joint tissue and cartilage degradation. The anti-RA effect of 15d-PGJ_2_ was associated with its ability to induce iTreg and to inhibit Th17 subset polarization.

It is well accepted that the presence of various proinflammatory cytokines in the joint environment contributes to the pathophysiology of autoimmune arthritis. Among these cytokines, IL-12, TNF-*α*, and IFN-*γ* play a central role in the RA pathology [2, 19]. In the last two decades, IL-17, a cytokine released mainly by Th17 cells, has gained importance as a cytokine that orchestrates arthritis pathology. For instance, the presence of IL-17 has been demonstrated in the synovial fluids and tissues of RA patients as well as in several experimental RA models. IL-17 mediates most RA events, including leukocyte recruitment to the joint as well as joint pain [20]. Moreover, IL-17 induces the release of several well-known proinflammatory cytokines, including TNF-*α* and chemokines [19]. The therapeutic treatment of the arthritic mice with 15d-PGJ_2_, administered daily subcutaneously for one week after the onset of disease, blocked the production of all abovementioned cytokines in the joint exudate. Furthermore, the presence of the ROR-*γ*t transcription factor, which is related to Th17 differentiation, was also inhibited in the draining lymph nodes by this treatment. Moreover, 15d-PGJ_2_ selectively suppressed effector cells, including Th17, as demonstrated* in vitro* by polyclonal (*α*-CD3) or specific (collagen-II) stimuli, blocking both IL-17 production and lymphocyte proliferation. Similarly, Klotz and coworkers have reported that pioglitazone, a synthetic PPAR-*γ* agonist, inhibits the differentiation of Th17 cells and thereby suppresses experimental autoimmune encephalitis [9]. In a murine model of allergic airway inflammation, the PPAR-*γ* agonists pioglitazone and rosiglitazone reversed the pathophysiological features of asthma by suppressing the release of IL-17 into the lung [12]. Regarding arthritis, at least to our knowledge, this is the first study to demonstrate the therapeutic effects of 15d-PGJ_2_ in a mouse model of rheumatoid arthritis. In rats with adjuvant-induced arthritis (AIA), pioglitazone decreased bone destruction by controlling the circulating and local expression of IL-17, with a subsequent decrease in the RANKL/OPG ratio [21]. We hypothesized that the potential mechanism by which 15d-PGJ_2_ decreased T cell proliferation could be mediated by inhibition of IL-2 secretion. Mechanistic studies indicate that PPARs intrinsically influence T helper differentiation and function and impair T cell proliferation through an IL-2 dependent mechanism involving repression of NFAT activity [7, 22]. Thus, the inhibition of the production of proinflammatory cytokines, especially IL-17, by 15d-PGJ_2_ treatment is likely a crucial step in limiting the tissue damage observed in RA. Moreover, it is reasonable to suggest that the inhibition of PPAR-*γ* may represent a new therapeutic strategy for RA.

The increased expression of PPAR-*γ* in arthritic was concomitant with ROR-*γ*t expression since such receptor is enhanced in most activated leukocytes, including Th17 subset, displaying a repressor role on inflammatory condition in promoting tissue repair and recovering the homeostasis [6]. Upon ligand binding, PPAR-*γ* heterodimerizes with the retinoid X receptor and binds to the PPAR response elements (PPRE) located in the promotor region of target genes [23, 24]. Additionally, the anti-inflammatory effects of PPAR-*γ* are mediated by negative pathway of proinflammatory cell signaling, for example, stabilization of corepressor complexes, such as nuclear corepressor (NCoR) and silencing mediator for retinoid and thyroid hormone receptors (SMRT) [23, 25]. Recently, it was described that ciglitazone inhibited both the proliferation of IL-17-producing cells and the expression of CCNB1, which regulates the cell cycle presumably by inhibiting cyclin B expression [26].

Although 15d-PGJ_2_ has high affinity for PPAR-*γ* and such receptor is highly expressed during activated phase of CIA, we not discard the possibility of 15d-PGJ_2_ to modulate the inflammation in PPAR-*γ*-independent mechanisms. Reports have shown that 15d-PGJ_2_ can repress some genes expression through the direct binding of ERK-MAPK and NF*κ*B [27, 28]. Furthermore, the proinflammatory production of NOS-2, metalloproteinase-2 (MMP-2), and MMP-9 as well as IL-6 and TNF-*α* in cultured cardiomyocytes infected with* Trypanosoma cruzi*, a protozoa parasite, was inhibited by administration of 15d-PGJ_2_, but rosiglitazone, a synthetic PPAR-*γ* agonist, was inefficient in inhibiting such mediators [29]. Up to date, there is no evidence of whether 15d-PGJ_2_ may selectively suppress Th17 cell differentiation in PPAR-*γ*-independent manner and the molecular mechanism remains to be investigated.

The anti-inflammatory activity of 15d-PGJ_2_ may be related to regulatory T cell generation. The levels of the classical transcription factor of regulatory T cells, FOXP3, were increased in the lymph nodes of treated animals. Several studies have reported that PPAR-*γ* agonists enhance the induction and function of Tregs in mice. Wohlfert and colleagues demonstrated that ciglitazone promoted the conversion of naïve T cells into CD4^+^FOXP3^+^ cells* in vitro *[30]. PPAR-*γ*-deficient Tregs exhibit an impaired ability to prevent effector T cells-induced colitis [31]. Furthermore, Iwami et al. showed that PPAR-*γ* agonists induce Tregs and prolong the survival of cardiac allografts [32]. Interestingly, we observed that the addition of 15d-PGJ_2_ into cell culture did not increase the numbers of nTregs (CD4^+^CD25^+^FOXP3^+^) but increased the levels of FOXP3, CTLA-4, and GITR in CD4^+^CD25^−^ cells, suggesting that the activation of PPAR-*γ* primarily induces the generation of iTregs. Furthermore, prostanoid treatment promoted a potent suppressive function* in vitro*. These findings are consistent with those obtained by Lei and colleagues, who demonstrated that PPAR-*γ* agonists, including 15d-PGJ_2_, induce and maintain FOXP3 expression in the CD4^+^CD25^−^ subpopulation of human lymphocytes [33]. Furthermore, iTreg can suppress the proliferation of effector T cells in a cell contact-independent fashion or through the production of anti-inflammatory cytokines such IL-10 [34, 35] and TGF-*β* [36]. Thus, expanding Treg cells during the ensuing chronic phase of disease may prevent collateral damage by suppressing the Th subset, especially Th17 cells. For unequivocal evidence of the effect of 15d-PGJ_2_ upon Treg generation and the suppression of Th17 cells, we submitted sorted naïve CD4^+^CD25^−^ cells to polarizing conditions for both subsets. The prostanoid treatment promoted Treg generation by increasing FOXP3 expression on CD4^+^CD25^−^ cells but restricted Th17 differentiation. Thus, the preservation of the joints of animals treated with prostanoid may be due to Treg cells.

Presumably, an aberrant Th17 response in the inflamed tissue may reflect Treg function during RA. Several studies have reported that Treg CD4^+^CD25^high^ cells are present in the inflamed synovium of arthritic patients and that their suppressive function is normal* in vitro *[37–39]. However, some studies have demonstrated that these cells exhibit a defect in their ability to suppress the proliferation of effector T cells present in the inflamed joint [37, 40]. Moreover, adoptive transfer of Treg cells may fail to heal well-established autoimmune diseases, indicating that, under certain chronic inflammatory conditions, Tregs are unable to mediate the immunosuppressive effect. Indeed, Tregs cultured with proinflammatory cytokines lose their capacity to inhibit responder cell proliferation and cytokine production [41]. Recently, our group demonstrated that the refractoriness of RA patients to methotrexate, the first-line pharmacotherapy for RA, is closely associated with a commitment to Treg expansion and function [42]. Thus, it is plausible to suggest that pharmacologic strategies that reinforce the suppressive function and/or the induction of Treg cells could represent a therapeutic target for the treatment of RA. Herein, we presented evidence that the blockage of Th17 with concomitant Treg generation by 15d-PGJ_2_ may be an interesting alternative to ameliorate the clinical symptoms of RA.

## 5. Conclusion

In the present study, we demonstrated that 15d-PGJ_2_ presents a potential therapeutic effect on collagen-induced rheumatoid arthritis (CIA). Such prostanoid suppresses the inflammatory process by promoting a regulator profile on T cells and in dampening the differentiation of Th17 cells. In last instance, it protect the articular joint from inflammatory insult.

## Supplementary Material

Supplementary material- Arthritic mice were treated by the subcutaneous route with vehicle or 15d-PGJ2 (1 mg/Kg) for 7 d and the mice were monitored for disease progression as indicated by clinical scores, number of affected paws, hypernociception and edema.

## Abbreviations

iTreg: Induced Treg
LN: Lymph node
nTreg: Natural Treg
Teff: Effector T cell
Treg: Regulatory T cell
WT: Wild type.
