# Supplementary material for: Therapeutic Treatment of Arthritic Mice with 15-Deoxy Δ12,14-Prostaglandin J2 (15d-PGJ2) Ameliorates Disease through the Suppression of Th17 Cells and the Induction of CD4+CD25−FOXP3+ Cells
Source: Mediators Inflamm. 2016 Oct 31;2016:9626427. doi: 10.1155/2016/9626427 (PMC5107840; doi:10.1155/2016/9626427)

Supplementary Table 1

Supplementary Table 1. Raw scores from arthritic mice

Days of treatment	Pain		Edema		Affected paws		Clinical score	
	Vehicle	PGJ2	Vehicle	PGJ2	Vehicle	PGJ2	Vehicle	PGJ2
1	4.16 ± 0.31	3.90 ± 0.65	1.97 ± 0.10	1.93 ± 0.02	1.2 ± 0.44	1.2 ± 0.44	1.2 ± 0.44	1.0 ± 1.34
2	4.42 ± 0.49	3.17 ± 0.64	2.21 ± 0.05	2.07 ± 0.12	2.4 ± 0.54	2.2 ± 0.83	5.8 ± 0.83	3.0 ± 1.22
3	5.12 ± 0.95	3.38 ± 0.66	2.26 ± 0.09	1.99 ± 0.09	2.4 ± 0.54	2.0 ± 0.70	6.0 ± 0.70	3.2 ± 0.83
4	5.55 ± 0.42	3.61 ± 0.61	2.27 ± 0.10	1.99 ± 0.12	2.4 ± 0.54	2.6 ± 0.54	6.0 ± 1.00	3.8 ± 0.83
5	6.20 ± 0.46	3.87 ± 0.99	2.35 ± 0.07	1.94 ± 0.12	2.4 ± 0.54	2.4 ± 0.54	6.0 ± 0.70	3.8 ± 0.83
6	6.06 ± 0.64	4.09 ± 0.90	2.33 ± 0.16	2.02 ± 0.11	2.4 ± 0.54	2.2 ± 0.44	6.2 ± 0.83	4.2 ± 0.83
7	6.22 ± 0.59	4.06 ± 0.59	2.29 ± 0.18	2.04 ± 0.11	2.6 ± 0.54	2.4 ± 1.14	6.6 ± 1.14	4.0 ± 1.00
8	6.13 ± 0.64	3.79 ± 0.70	2.38 ± 0.17	2.11 ± 0.17	2.4 ± 0.54	2.4 ± 0.89	6.6 ± 1.34	4.2 ± 1.30

Table Legend: Arthritic mice were treated by the subcutaneous route with vehicle or 15d-PGJ_2 (1 mg/Kg) for 7 d and the mice were monitored for disease progression as indicated by clinical scores, number of affected paws, hypernociception and edema Data are expressed as the mean ± SEM.

Supplementary Figure 1

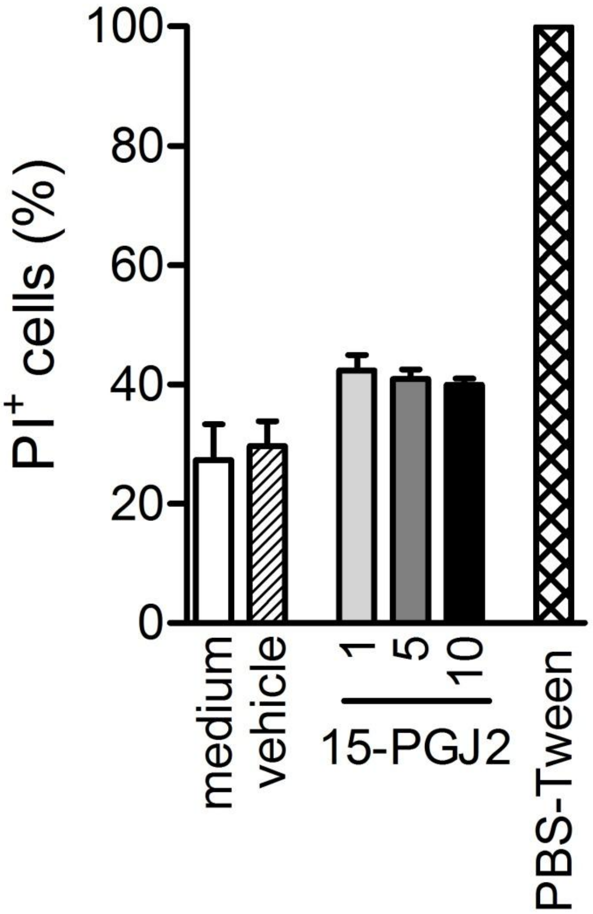

Supplement: Supplementary file 1 — Supplementary material- Arthritic mice were treated by the subcutaneous route with vehicle or 15d-PGJ2 (1 mg/Kg) for 7 d and the mice were monitored for disease progression as indicated by clinical scores, number of affected paws, hypernociception and edema. [file 9626427.f1.pdf]
